# Enforced home-working under lockdown and its impact on employee wellbeing: a cross-sectional study

**DOI:** 10.1186/s12889-022-12630-1

**Published:** 2022-01-29

**Authors:** Katharine Platts, Jeff Breckon, Ellen Marshall

**Affiliations:** 1 Advanced Wellbeing Research Centre, Sheffield Hallam University, Olympic Legacy Park, Sheffield, S9 3TU UK; 2 Academy of Sport & Physical Activity, Sheffield Hallam University, Collegiate Campus, Sheffield, S10 2BP UK; 3 Department of Engineering and Maths, Sheffield Hallam University, City Campus, Sheffield, S1 1WB UK

**Keywords:** Home-working, Mental health, Wellbeing, Stress, Work-life conflict, Leadership

## Abstract

**Background:**

The Covid-19 pandemic precipitated a shift in the working practices of millions of people. Nearly half the British workforce (47%) reported to be working at home under lockdown in April 2020. This study investigated the impact of enforced home-working under lockdown on employee wellbeing via markers of stress, burnout, depressive symptoms, and sleep. Moderating effects of factors including age, gender, number of dependants, mental health status and work status were examined alongside work-related factors including work-life conflict and leadership quality.

**Method:**

Cross-sectional data were collected over a 12-week period from May to August 2020 using an online survey. Job-related and wellbeing factors were measured using items from the COPSOQIII. Stress, burnout, somatic stress, cognitive stress, and sleep trouble were tested together using MANOVA and MANCOVA to identify mediating effects. T-tests and one-way ANOVA identified differences in overall stress. Regression trees identified groups with highest and lowest levels of stress and depressive symptoms.

**Results:**

81% of respondents were working at home either full or part-time (*n* = 623, 62% female). Detrimental health impacts of home-working during lockdown were most acutely experienced by those with existing mental health conditions regardless of age, gender, or work status, and were exacerbated by working regular overtime. In those without mental health conditions, predictors of stress and depressive symptoms were being female, under 45 years, home-working part-time and two dependants, though men reported greater levels of work-life conflict. Place and pattern of work had a greater impact on women. Lower leadership quality was a significant predictor of stress and burnout for both men and women, and, for employees aged > 45 years, had significant impact on level of depressive symptoms experienced.

**Conclusions:**

Experience of home-working under lockdown varies amongst groups. Knowledge of these differences provide employers with tools to better manage employee wellbeing during periods of crisis. While personal factors are not controllable, the quality of leadership provided to employees, and the ‘place and pattern’ of work, can be actively managed to positive effect. Innovative flexible working practices will help to build greater workforce resilience.

## Background

The Covid-19 pandemic, and subsequent public health lockdowns around the world, have precipitated a shift in the working practices of millions of people. An estimated 47% of the British workforce reported to be working at home in April 2020 (compared to 5% in 2019), with 86% of this number a direct result of the Covid-19 national lockdown [1].

Home-working, or ‘home-based telework’ as it is sometimes termed [2], has traditionally been undertaken by mutual agreement between employer and employee, typically in white-collar and professional occupations. Most of what is known about the impact of home-working on employees is in the context of voluntary and consensual arrangements, such as flexible working schedules and hybrid arrangements where time is shared between remote telework and office-based work. How a sudden and unexpected change in working circumstances impacts the psychological, emotional, and physiological wellbeing of workers is not well understood, and yet there is broad consensus that positive employee wellbeing is an important precursor to positive performance at work [3].

### Conceptualization of wellbeing at work

Work-related wellbeing as characterised by Van Horn et al. [4] comprises the five interrelated dimensions of *affective wellbeing* (mood/affect, job satisfaction, organisational commitment, emotional exhaustion); *cognitive wellbeing* (cognitive weariness, concentration and taking up new information); *social wellbeing* (social functioning in relationships with colleagues); *professional wellbeing* (autonomy, aspiration, and competence); and *psychosomatic wellbeing* (physical health). This multi-dimensional approach provides a broader frame of reference to help understand the organisational and job-related factors that influence personal wellbeing. Though focused on the individual, these constructs are important to employers who must ensure that gains are not achieved at the cost of poor employee health outcomes [5].

### Wellbeing in a home-working context

Experience of home-working will differ from population to population. While many studies have supported the view that home-working engenders positive health outcomes such as reduction in stress [6–9], burnout [10] and fatigue [11, 12], as well as increases in general happiness [11] and quality of life [8, 13], others have found detrimental impacts to general psychological wellbeing [14, 15], burnout [16], and work-life balance [17, 18]. Nevertheless, the mechanisms driving these effects are not always clear and are dependent upon a range of individual and environmental factors. Gender and parental status, for example, play key roles in the nature and experience of working at home, as this arrangement tends to promote a more traditional division of labour, with women often using home-working as a tool to maintain work capacity in periods of increased family demands, such as after childbirth [19].

Flexible working arrangements, including working at home, that increase employee autonomy and choice are generally found to be conducive to positive wellbeing [20] and may help improve work-life balance. Flexible working arrangements are more accommodating of individual needs and allow for greater employee independence, higher levels of work-time control and agency over work-related decisions (autonomy), yet are associated with significantly higher levels of work-life conflict [21], where work concerns distract from and disrupt home life (or vice versa) wherein stress is induced or increased and efforts at sleep and recovery are hampered [22, 23].

### The elimination of choice – transitioning to a an ‘enforced’ home-working scenario

Inquiry into whether what is known about home-working under ‘normal’ circumstances holds true when the element of personal choice is removed, and the worker may have to share space and resources with other household members mandated to stay at home under lockdown.

Recent research suggests that mandated home-working environments may have negative impacts from a physical health perspective [24], that the persistent overuse of technology for communications is increasing levels of stress [25], and the social deficit created by lack of interpersonal contact while working at home under lockdown may be detrimental to emotional wellbeing [26]. Some have suggested that work-life conflict is exacerbated in an environment where the boundaries between work and home are permeable and ill-defined, in particular at a time when leaving the home for long periods of time is not possible, such as during lockdown [18]. For employees managing long-term mental health conditions, working at home during lockdown is likely to have had serious negative consequences, as routines are disrupted and access to critical support services and social contact are lost [27, 28].

Female employees may be more at risk of emotional exhaustion and physical health problems under lockdown circumstances than male employees [29, 30], and increased autonomy, such as the ability to adjust working times and work overtime to catch up on work at home, has a particularly detrimental impact on women due to increased work-life conflict [31]. Women, and those of both genders in younger age groups (< 35 years), more often report high emotional demands at work and physical exhaustion during periods of mandated home-working [32].

Quality of leadership, supervisory and collegial support all influence employee experience, yet in a time of crisis such as the Covid-19 pandemic lockdown, organisational leaders may not be properly equipped to manage their people from a distance, lacking the essential skills of effective ‘virtual leaders’ [33, 34].

### Aims of this study

This study examined the combined impact of age, gender, dependants, mental health status and work status in relation to enforced home-working and the effects on wellbeing markers including stress, burnout, depressive symptoms, and sleep in UK employees. The study considered the following across public, private and third sector organisations; (i) which groups have the poorest wellbeing levels at a time of mandated home-working and (ii) which factors exert significant moderating and mediating influences; both in terms of personal and environmental factors such as gender, age and dependants, and work-related factors such as quality of leadership and social support.

## Methods

### Participants and procedures

Ethical approval for the study was obtained via Sheffield Hallam University Research Ethics Committee (No. ER23891582). Private, public and third sector organisations operating in the United Kingdom were invited to participate in the study. Participating organisations were required to have a significant proportion of their workforce involuntarily working from home due to Covid-19 pandemic lockdown measures. Nine organisations volunteered to participate, with private (*n* = 5), public (*n* = 2) and third sector (*n* = 2) organisations represented in the sample. A total of 623 adults from these organisations responded to an invitation to participate delivered via their employer. Individual participant inclusion criteria included being of working age (18 years +) and in either full-time or part-time employment. A summary of participant demographics can be found in Table 1.

**Table 1:** Stress factor and mean values for depressive symptoms and work-life conflict by age, gender, mental health status and number of dependants

	**Category**	**N**	**%**	**Stress factor (M,SD)**	**Depressive symptoms (M,SD)**	**Work-life conflict (M,SD)**
All participants		623	100%	0 (1)	29 (22.6)	30 (23)
Age	16–24	30	5%	0.04 (0.9)	41 (21.4)	22 (15.8)
	25–34	135	22%	0.21 (1)	36 (24.8)	33 (21.5)
	35–44	149	24%	0.2 (1)	32 (22.6)	36 (24.6)
	45–54	200	32%	-0.19 (0.9)	25 (21.2)	27 (23.1)
	55 +	109	17%	-0.21 (0.9)	24 (19)	27 (22.4)
	*ANOVA TS and p-value*			*F* = *5.74, p* < *0.001*	*F* = *8.98, p* < *0.001*	*F* = *4.9, p* = *0.001*
Gender	Male	234	38%	-0.16 (0.91)	24 (20.2)	33 (22.1)
	Female	384	62%	0.1 (1.03)	32 (23)	29 (23.5)
	*Missing*	*5*	1%			
	*Independent t-test (TS, p-value)*			*t* = *-3.06, p* = *0.002*	*t* = *-4.19, p* < *0.002*	*t* = *2.31, p* = *0.021*
Diagnosed mental health condition	No	537	86%	-0.1 (0.93)	27 (20.8)	30 (22.8)
	Yes	68	11%	0.83 (1.11)	47 (27.5)	34 (24.6)
	*Missing*	*18*	3%			
	*Independent t-test (TS, p-value)*			*t* = *-7.5, p* < *0.001*	*t* = *-5.7, p* < *0.001*	*t* = *-1.29, p* = *0.199*
No. of dependants	0	293	47%	-0.1 (1)	30 (23.9)	25 (22.3)
	1	125	20%	-0.04 (1)	29 (22.6)	30 (21.1)
	2	133	21%	0.27 (1)	31 (20.8)	42 (24.3)
	3 +	72	12%	-0.02 (0.9)	27 (20.4)	30 (19.5)
	*ANOVA TS and p-values*			*F* = *4.24, p* = *0.006*	*F* = *0.4, p* = *0.753*	*F* = *15.8, p* < *0.001*

*M* mean score 0–100, *SD* Standard Deviation

### Data collection and measures

Cross-sectional data were collected over a 12-week period from May 2020 to August 2020 during the first wave of Covid-19 pandemic lockdown measures in the UK. A 33-item questionnaire was developed for the purposes of the study and delivered online using Qualtrics secure web-survey (© Qualtrics LLC, 2021). Informed participant consent was collected on the Qualtrics platform prior to data collection, and those that did not consent were not able to access the survey.

Five demographic items were collected: age category, gender, number of dependants, mental health status (defined in two categories as presence or absence of diagnosed mental health condition), and work status (defined in four categories as working at home full-time, working at home part-time, working in usual place of work, furloughed).

Job-related and health and wellbeing factors were measured using 28 items from the English version of the Third Copenhagen Psychosocial Risk Assessment Questionnaire (COPSOQIII) [35] comprising core items plus additional items from the middle and long version as appropriate. The COPSOQIII was deemed appropriate for the study due to its effectiveness across diverse industry sectors and in organisations of varying sizes, and for allowing analysis against different workplace wellbeing frameworks including the Five-Dimension Model [4].

Work-related factors in six domains were investigated by assessing to what extent respondents were able to exert control over breaks (1 item), extent of overtime worked (1 item), how they rated quality of organisational leadership (2 items), how they rated social support from their supervisor and colleagues (2 items) and level of work-life conflict experienced (4 items). Work-related items were measured on a 5-point rating scales using various statements appropriate to the question.

Wellbeing factors in six domains were investigated by assessing to what extent the respondent suffered from common symptoms. The domains were sleeping troubles (1 item), burnout (4 items), stress (3 items), somatic stress (3 items), cognitive stress (3 items) and depressive symptoms (4 items). Wellbeing items were measured on a 5-point rating scale (scored as 100 = all the time, 75 = a large part of the time, 50 = part of the time, 25 = a small part of the time, 0 = not at all). All wellbeing subscales showed good internal consistency (Cronbach’s $\alpha >0.8$) but the two items from the ‘control over working time’ subscale (control over breaks and extent of overtime worked) had poor consistency and were therefore used separately in analysis.

### Data analysis

Statistical analyses of data were undertaken using IBM Statistical Package for the Social Sciences (SPSS Version No. 26) Stress, burnout, somatic stress, cognitive stress and sleep trouble were all at least moderately correlated and demonstrated similar impact so were tested together using MANOVA initially and then MANCOVA to test mediating effects, all with Tukey post-hoc tests; whereas group comparisons for depressive symptoms were not consistent and were tested separately for all analyses.

A standardised factor score to represent ‘overall stress’ (alpha = 0.87) was created from the stress-related subscales stress, burnout, somatic stress, cognitive stress, and sleep trouble, and used instead of the individual variables. Initial analysis used independent t-tests and one-way ANOVA to test differences in overall stress, for each of the key demographic variables. Regression trees were used to identify groups with higher levels of the stress factor score or depressive symptoms using the core demographic variables of interest and the key work-related factors of quality of leadership, social support, and work-life conflict.

## Results

A total of 623 people completed the survey (62% female). 53% of all participants had one or more dependants, while 11% reported a diagnosed mental health condition. The majority (81%) were working at home because of lockdown restrictions, either full-time or part-time, while 9% continued to work in their usual place. 5% of participants were furloughed and so did not complete all the questions from the work-related subscales. 5% of people defined their work status as ‘other’ and were removed from analyses where work status was considered.

### Gender, age, mental health status, dependants and work status effects on wellbeing markers and work-life conflict

As shown in Table 1, women had significantly higher levels of stress and depressive symptoms (t = -3.06, *p* = 0.002; t = -4.19, *p* < 0.002), but men reported significantly higher levels of work-life conflict (t = 2.31, *p* = 0.021). Across both genders, those aged 25–44 years had significantly higher stress compared to those aged 45 + years (*F* = 8.98, *p* < 0.001). Depressive symptoms decreased with age, with those aged 16–24 years reporting the highest levels, and those aged 45 + years reporting lower levels than all other age groups. The 35–44 age group reported significantly higher levels of work-life conflict than those aged < 25 years or 45 + years of age (*F* = 4.9, *p* = 0.001). Those who reported a diagnosed mental health condition had significantly higher stress and depressive symptoms than those who did not (t = -7.5, *p* < 0.001; t = -5.7, *p* < 0.001), but no significant difference in work-life conflict was found between these two groups. The number of dependants did not impact on depressive symptoms, but stress variables were found to be consistently higher for those with two dependants (*F* = 4.24, *p* = 0.006). Levels of work-life conflict were significantly higher for those with two dependants when compared to the effects of 0, 1, or 3 + dependants (*F* = 15.8, *p* < 0.001).

**Fig. 1:**
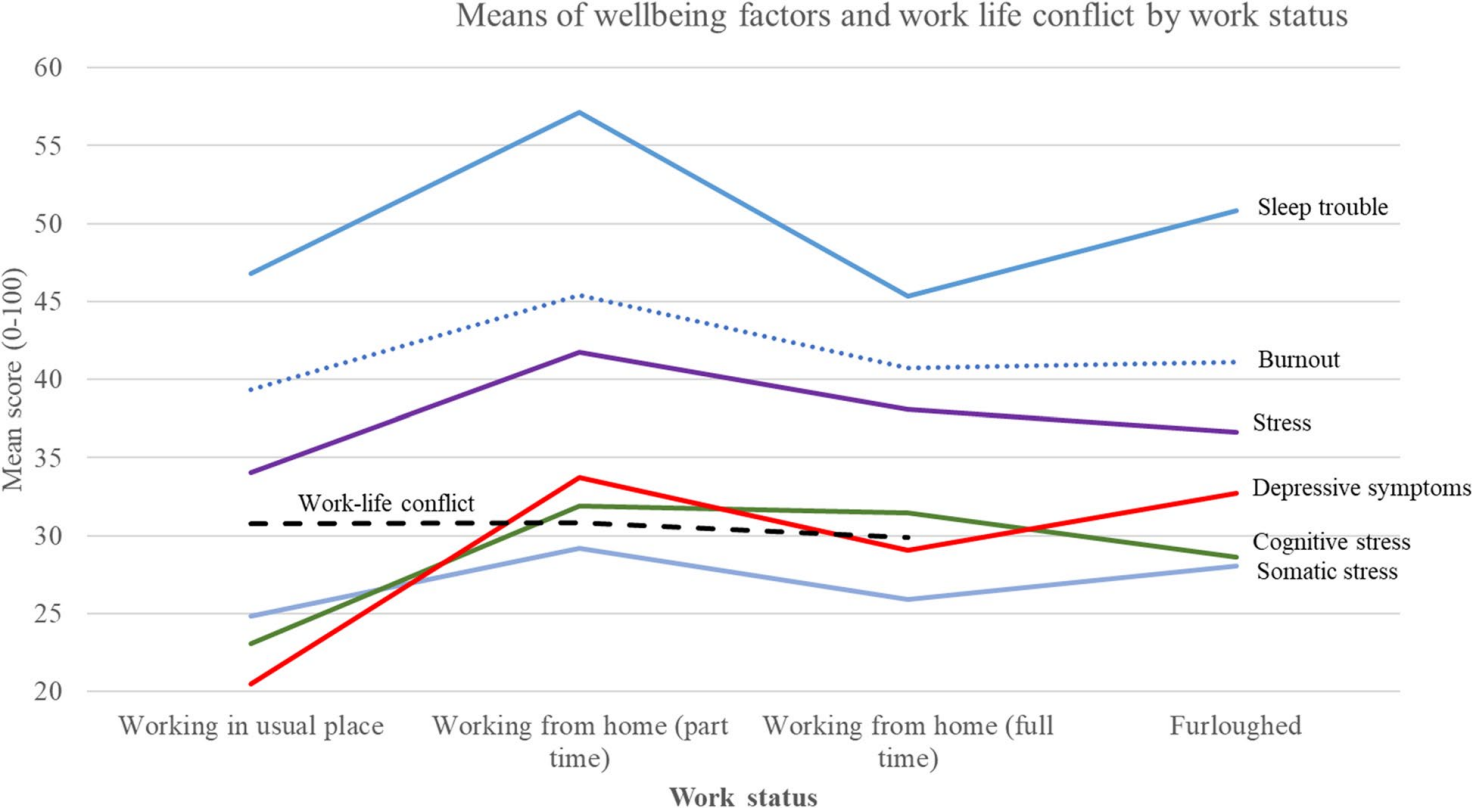
Wellbeing factors and work life conflict by work status

As shown in Fig. 1, those working at home part-time generally had the highest levels of stress and depressive symptoms. There were significant work status differences for sleeping troubles (*F* = 5.32, *p* = 0.001), with those working at home part-time having significantly higher levels of sleep troubles than those working at home full-time. Those working at home full-time or part-time, and those furloughed, had significantly higher levels of depressive symptoms than those working in their usual place of work (*F* = 3.94, *p* = 0.009).

### Work status and mental health

**Fig. 2:**
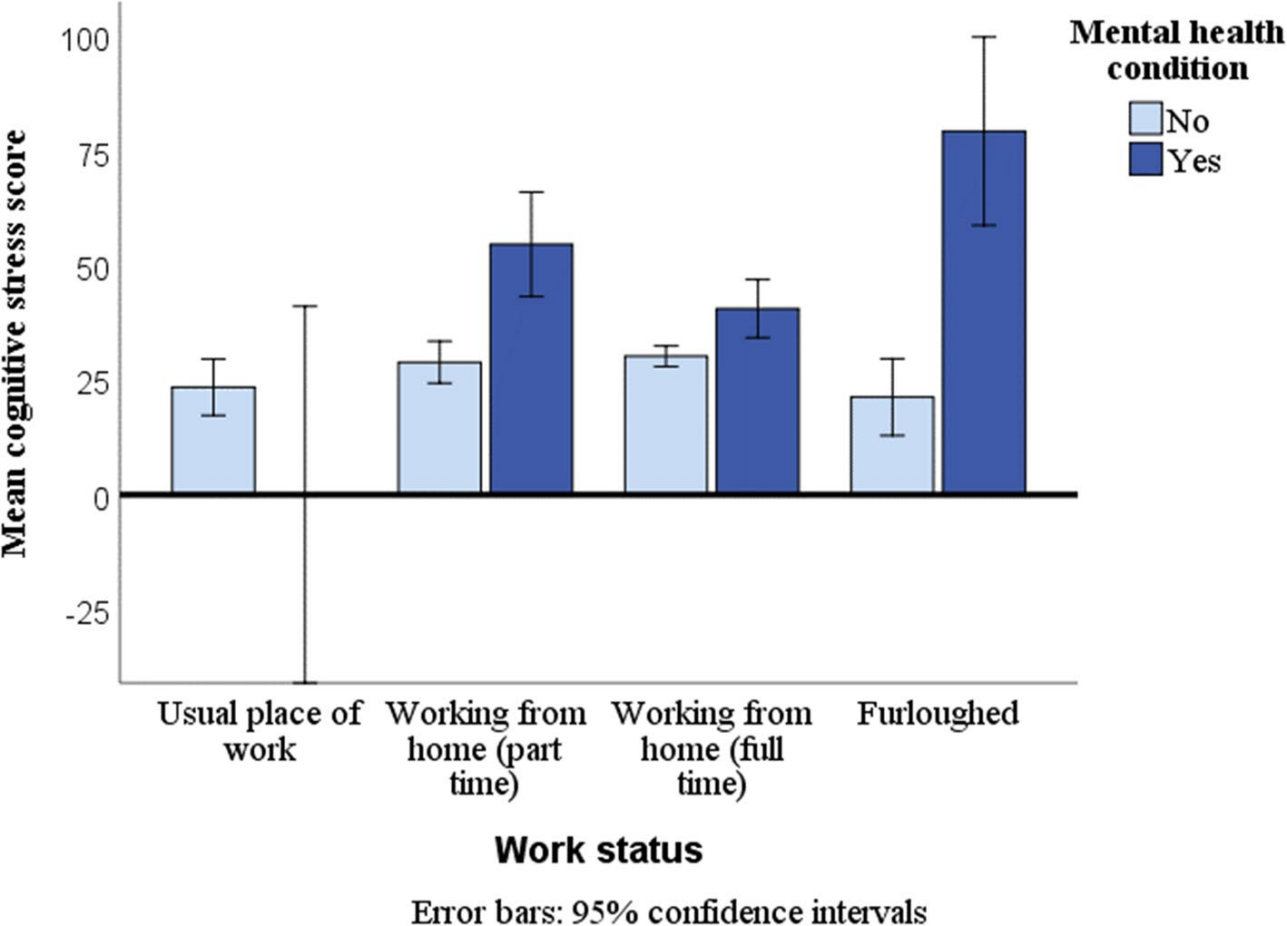
Mean cognitive stress (marginal) by work status and mental health status. 95% Confidence Intervals

There was a significant interaction between work status and mental health [Wilk's Λ = 0.935, *p* = 0.002, ${{\eta }_{p}}^{2}=0.022]$ for the stress-related variables, and for depressive symptoms [*F*(3,534) = 3.35, *p* = 0.019, ${{\eta }_{p}}^{2}=0.018]$. Those with a diagnosed mental health condition had consistently higher levels of stress, cognitive stress, somatic stress, burnout, and sleep troubles when working at home or furloughed. Within this group, part-time home-workers and those who were furloughed experienced the highest levels stress and depressive symptoms (see Fig. 2 as a typical example), although the differences were not significant for depressive symptoms.

### Combined effects of work status and gender

**Fig. 3:**
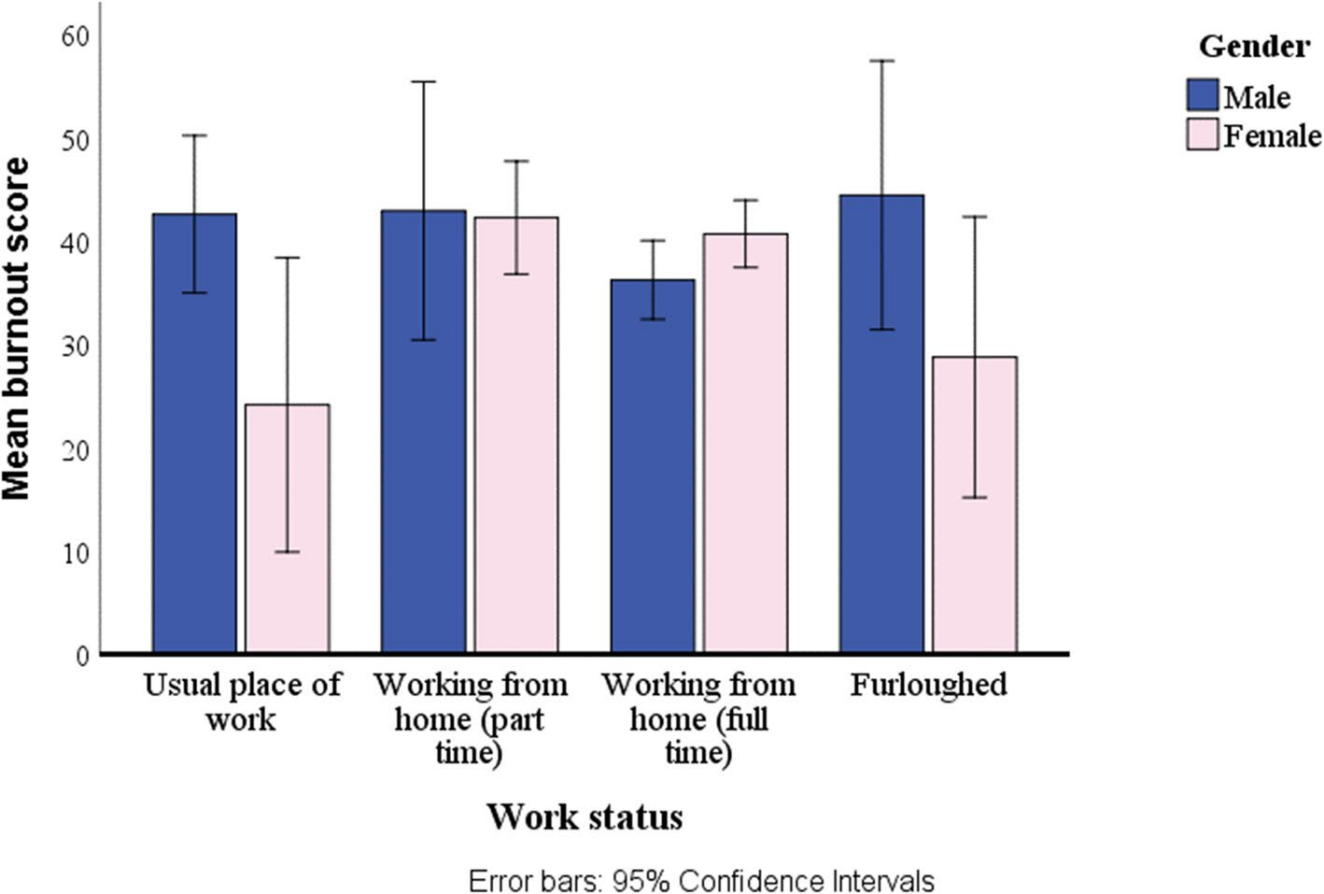
Burnout by work status and gender

The combined effects of work status and gender were only examined for the group with no diagnosed mental health condition. The interaction was significant between work status and gender for stress [Wilk's Λ = 0.935, *p* = 0.007, ${{\eta }_{p}}^{2}=0.022]$. As shown in the example in Fig. 3, work status generally had more of an impact on women, with those working at home, particularly part-time, having consistently higher scores on the stress variables. For those in their usual place of work, men scored significantly higher than women for stress and burnout.

Quality of leadership was a significant negative predictor of stress, burnout, somatic and cognitive stress for both men and women [Wilk's Λ = 0.959, *p* = 0.003, ${{\eta }_{p}}^{2}=0.041]$. After controlling for quality of leadership, the interaction between gender and work status for stress was still significant [Wilk's Λ = 0.951, *p* = 0.015, ${{\eta }_{p}}^{2}=0.025]$ but the place-of-work differences observed for women were reduced, so quality of leadership was not a mediating factor.

After controlling for work-life conflict, the interaction between gender and work status for stress was significant [Wilk's Λ = 0.953, *p* = 0.017, ${{\eta }_{p}}^{2}=0.024]$], and gender differences increased as men had higher work-life conflict scores generally. For those working full-time at home, women had significantly higher stress, burnout, somatic stress, and sleep trouble than men after controlling for work-life conflict. Women working at home part-time had significantly higher stress scores than men, and women in their usual place of work had significantly higher levels of sleep troubles. No significant interactions were found between work status and gender for depressive symptoms.

### Combined effects of work status and age

**Fig. 4:**
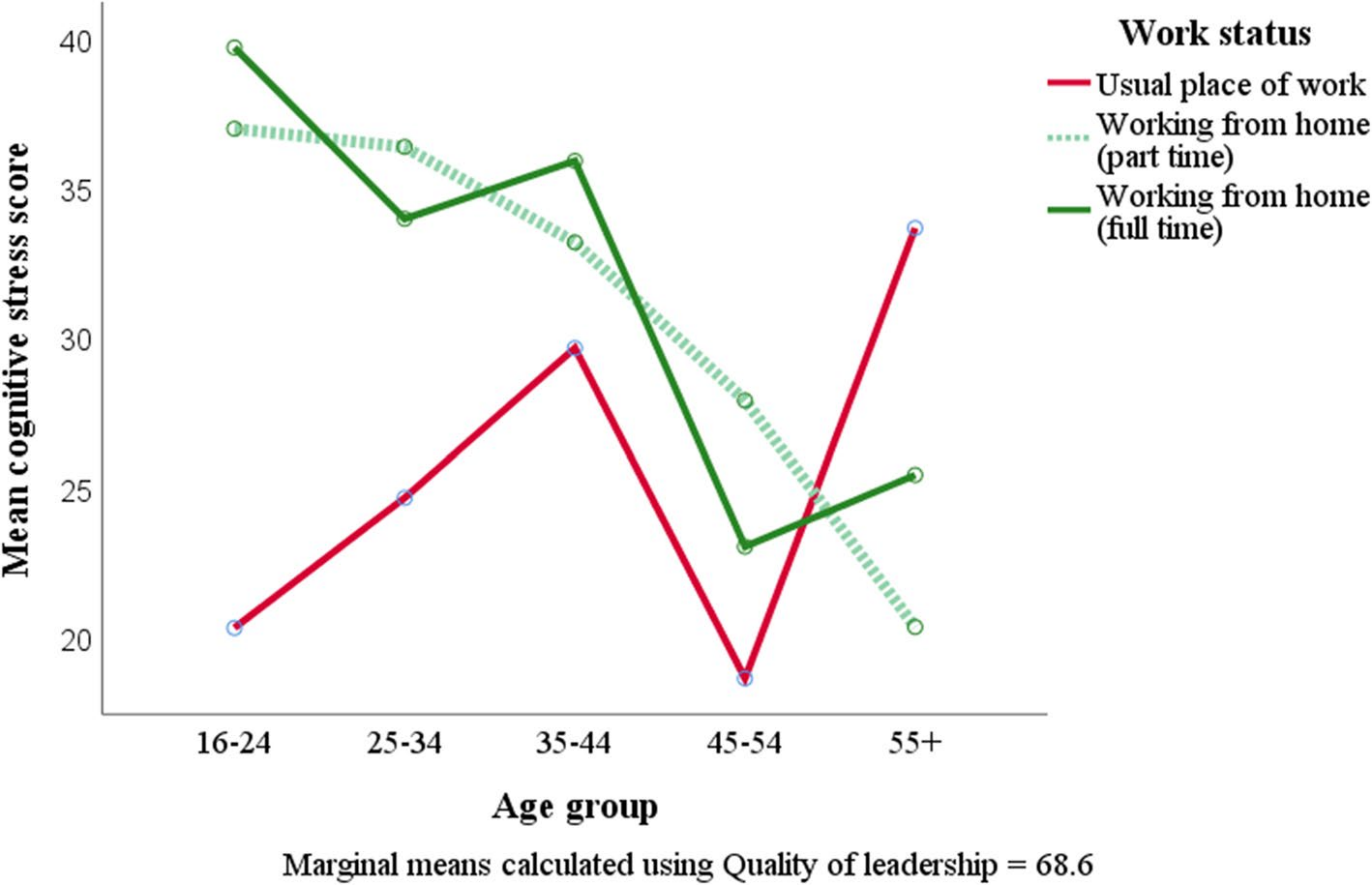
Cognitive stress by work status and age group

The interaction between work status and age group for stress-related factors was significant (Wilk’s lambda = 0.848, *p* = 0.037, ${{\eta }_{p}}^{2}=0.034$) for the group with no diagnosed mental health condition, with age impacting most on part-time home-workers, and those in the 35–44-year age group most stressed. After controlling for quality of leadership, differences became more pronounced and a more general downward trend by age group was observed – particularly for somatic and cognitive stress (Fig. 4). No significant interactions were found between work status and age for depressive symptoms.

### Combined effects of work status and number of dependants

The interaction between work status and number of dependants for stress was not significant so was removed from the model. The interaction between work status and number of dependants for depressive symptoms was borderline significant [F(9,466) = 800.9, *p* = 0.054,,${{\eta }_{p}}^{2}=0.035$]. Those with one dependant whilst working at home full-time or part-time had significantly higher levels of depressive symptoms than those in their usual place of work. Those with 3 + dependants and on furlough leave experienced significantly more depressive symptoms than those in their usual place of work.

### Identifying groups of workers with highest and lowest levels of depressive symptoms or stress.

**Fig. 5:**
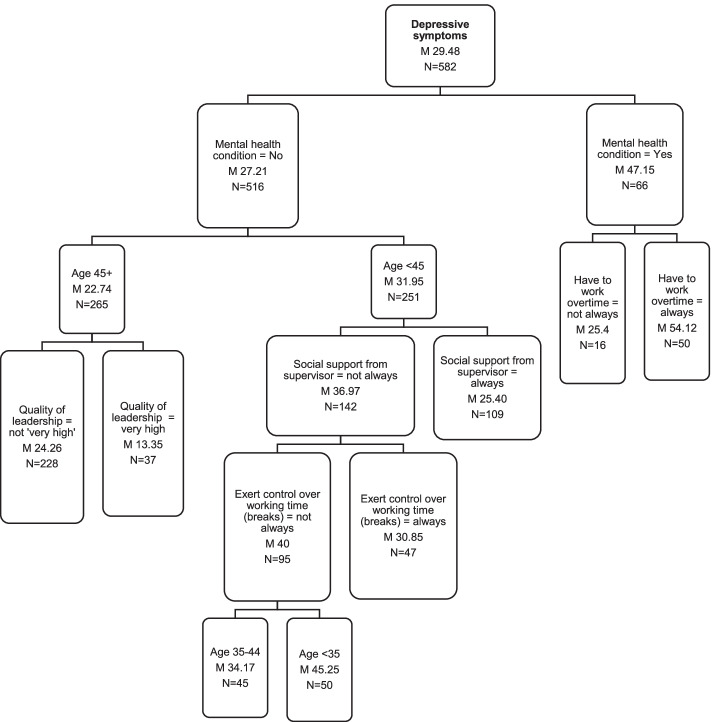
Comparison of wellbeing and work-related factor means by depressive symptoms regression tree group (M = mean score 0–100)

Regression tree analysis enabled further groupings to be identified from a wider range of variables. Figure 5 shows the regression tree with depressive symptoms as the dependent variable and all key demographic and work-related factors included. The presence or absence of a mental health condition gave rise to the largest difference in mean level of depressive symptoms (20-point difference), so the groups were separated first. Those with an existing mental health condition who occasionally or always worked overtime were the most depressed group (M 54.1, 95% CI 47,61). Those with a mental health condition who never worked overtime had a much lower score for depressive symptoms (M 25.4, 95% CI 14,37) which is more in line with the group with no mental health condition (M 27.2).

Amongst those with no mental health condition, the sub-group with the lowest depressive symptoms overall was the one aged over 45 years who rated quality of leadership highly (M13.35, 95% CI 9,18), and the sub-group with the highest levels of depressive symptoms overall were those aged under 35 years, who didn’t always have social support from their supervisor and could not always exert control over taking breaks (M 45.3, 95% CI 39,52).

Ten sub-groups were identified using the standardised stress factor score as the dependent variable which has a mean of zero; thus, positive scores were above average and negative scores were below average. Table 3 shows the group means for each of the stress variables as well as the standardised score for each group. The presence or absence of a mental health condition gave rise to the largest difference in mean level of stress, so those groups were separated first. Mean stress levels were highest overall in the sub-group with diagnosed mental health conditions that always had to work overtime (M 1.16, 94% CI 0.89,1.42). For the group with no mental health condition, the key variables selected to separate groups were quality of leadership, age, control over breaks, number of dependants and gender. Where quality of leadership was low (M < 69), the group with the highest mean levels of stress were those aged 25–44 (M 0.41, 95% CI: 0.21,0.62). Where quality of leadership was high (M 69 +) the group with the highest levels of stress had 2 + dependants and were less able to exert control over breaks (M 0.57, 95% CI: 0.15,1). This sub-group also had the highest levels of burnout, somatic stress, and sleep trouble. The sub-group with the lowest levels of stress were those aged under 25 or 45 + who had high levels of control over their breaks and zero dependants (M -0.97, 95% CI: -1.3,-0.64). This group also had the lowest levels of burnout, somatic stress, cognitive stress and depressive symptoms and relatively high levels of social support.

**Table 3:** Comparison of wellbeing and work-related factor means by overall stress regression tree group

Presence of mental health condition		Yes		No mental health condition							
Must work overtime		Never	Any								
Quality of leadership				< 69				Quality of leadership 69 +			
Age group				25–44	< 25, 45 +						
Control over working time (breaks)					Not always	Always			Never/Sometimes	Often/always	
No. of dependants						0	1 +	0, 1	2 +		
Gender										Male	Female
Size of group (N)	582	16	50	96	84	20	23	195	16	40	42
	Overall mean	Group means									
Overall stress and sleep factor	0	-0.21	1.16	0.41	0.08	-0.97	-0.12	-0.41	0.57	-0.33	0.12
Burnout	42	36	65	51	44	17	42	33	53	35	42
Stress	39	30	59	49	42	19	36	29	49	33	41
Somatic Stress	26	24	47	31	28	13	22	20	35	21	30
Cognitive Stress	31	20	52	38	30	11	29	24	36	27	33
Sleep trouble	48	59	73	54	47	35	46	40	69	38	49
Depressive symptoms	29	25	54	38	31	8	20	23	31	19	33
Work-Life conflict	30	18	39	39	37	14	30	22	47	29	33
Social support—supervisor	79	83	78	62	64	70	61	90	91	92	90
Social support—colleagues	79	89	76	73	74	76	68	83	84	83	89
Quality of leadership	68	68	67	47	47	44	47	84	81	86	86
Control over working time (breaks)	80	89	80	78	66	100	100	82	38	87	90
Must work overtime	40	0	48	43	39	18	36	39	57	47	37

## Discussion

Employee wellbeing has been impacted by the recent global pandemic, typically resulting from increased levels of enforced home-working. This study set out to examine the impact of age, gender, dependants, mental health status and work status on employee wellbeing under enforced home-working conditions, as well as the influence of work-related factors such as work-life conflict, quality of leadership and social support from supervisors and colleagues.

The findings suggest that detrimental wellbeing impacts of enforced home-working are most acutely experienced by those with existing mental health conditions, regardless of age, gender, or work status, and that home-working and having to work regular overtime strongly exacerbate issues of poor sleep, stress, and depression in those who are suffering with mental health issues. In healthy individuals, both age and gender appear to play moderating roles in feelings of stress and depression at times of enforced home-working, with women and younger age groups generally faring worse than others.

Working pattern and place (‘work status’) has emerged [36], alongside the presence of a mental health condition, as a key factor in determining wellbeing impacts of enforced home-working, with place and pattern of work having a greater impact on women. Those working at home full- or part-time reported significantly higher levels of stress and depression than those who continued to work in their usual place during lockdown, indicating that abrupt disruption to routine and unfamiliarity of working practices and environment, potentially coupled with job insecurity and concerns about the pandemic in general, has a broadly negative effect on emotional wellbeing.

Quality of leadership and social support from colleagues also play key roles in moderating wellbeing outcomes, with leadership quality particularly influential in mental health outcomes for younger age groups. Poor organisational leadership and the requirement to work after hours are known to be significantly associated with occupational stress, anxiety, and depression [37, 38] and for those with mental health issues these factors appear amplified; indeed, where regular overtime is not required, the positive impact on depressive symptoms in this cohort is considerable. Where leadership quality was rated highly in the present study, it had a positive impact by reducing stress and depressive symptoms in those working at home full-time with a diagnosed mental health condition. This reinforces the critical role organisational leaders play in mitigating any damaging effects of home-working in those suffering poor mental health, and thus should be a priority for organisations.

Any individual may suffer altered mood states on a short-, medium- or long-term basis which are experienced as depressive symptoms, stress, and poor sleep, as has been the case for much of the global population during the Covid-19 pandemic [39]. In the UK, around 1 in 5 adults reported feelings of depression in early 2021 – over double pre-pandemic levels [40]. In the present study, leadership quality impacted across several healthy groups and influenced the extent to which employees experienced stress, depressive symptoms and trouble sleeping. For example, leadership quality strongly influenced experience of depressive symptoms in employees aged 45 + , with those experiencing ‘very high’ leadership quality suffering virtually no depressive symptoms at all, compared to those who were not. This evidence suggests that the most important protective factors against stress and depressive symptoms were not having an existing mental health condition and high quality of leadership, the latter of which may act, for example, as a buffer against the stresses of a lack of work resources [41].

### The role of gender and work status on mental health

Women’s psychological health appears to have been deeply affected by the pandemic [42]. Women have suffered significant and clinically relevant declines in mental wellbeing [39] alongside generally higher levels of health anxiety [43]. Evidence from this study shows that women suffered higher levels of stress, burnout, somatic stress, sleep trouble and depressive symptoms than their male counterparts during lockdown, particularly when home-working on a part-time basis, while men reported higher levels of work-life conflict.

As many organisations consider a move to permanent remote-working or ‘hybrid’ working models in the wake of the pandemic, they must be appropriately sensitive to the mental health challenges this may bring about for working women. Approximately 70% of the British national part-time workforce are women (some 5.67 million women in Q1 2021) [44] and the choice of many women to work part-time appears to be connected to childcare responsibilities [45]. Childcare and housework responsibilities remain predominantly within the remit of the mother (in households with children), with women in part-time work spending more time on house-work and childcare than those in full-time work [46]. Women working from home during lockdown with no access to supportive childcare are especially exhausted [42]. It is feasible that long periods of involuntary part-time home-working, such as that which could be imposed via a ‘hybrid’ model, could results in increased poor health outcomes for women as they struggle to balance domestic and professional responsibilities.

The impacts of enforced (often abrupt) new working patterns and practices appear to be equally felt by men. Working parents in general have higher levels of stress [47] and work-life conflict [48], and this study found that overall stress was significantly higher for individuals of either gender with two dependants (compared to 0,1, or 3 + dependants), although no impacts on depressive symptoms were found. Therefore, while women report more negative psychosomatic wellbeing effects, men appear to experience the greatest disruption under lockdown, reporting the highest levels of work-life conflict while home-working – which was itself observed to have a strong positive relationship with stress. This finding is somewhat unexpected and suggests that women are in some way better prepared to manage disruptions to their working life than men, which may be due to persisting traditional gender and parenting roles. The presence of dependants at home and age of dependants will influence stress-related issues [48], so in the absence of physical or temporal boundaries between work and home life, how effective an individual is at managing their transition between work and non-work activity whilst home-working may strongly influence the level of work-life conflict they experience [49], regardless of gender.

### The influence of age and work status on mental health

For young adults in the UK, experience of depressive symptoms more than doubled during the pandemic, with 29% of those aged 16–39 reporting symptoms in early 2021 [40]. The reasons underpinning this wave of poor mental health are complex, but loneliness, work uncertainty, and financial insecurity are all indicated as factors that have amplified feelings of depression and sadness in young people during the pandemic [49–51].

For individuals without diagnosed mental health conditions, age emerges in this study as the key variable in determining level of depression and stress during periods of enforced home-working, with symptoms of both decreasing with age. After controlling for quality of leadership, differences between age groups became more pronounced and a downward trend by age was observed – particularly for somatic and cognitive stress. With poor ‘*cognitive wellbeing’* [4] comes lack of concentration, weariness, and burnout [52], yet a simple change in schedule may decrease the likelihood of job stress by 20% and increase job satisfaction [53] providing further evidence of the importance of competent and ‘health promoting’ leadership to maintain both positive wellbeing [54] and work engagement.

Professional isolation and lack of contact and communication with colleagues will negatively affect mental wellbeing in times of home-working during a crisis [55, 56]. In this study, those under 35 without a pre-existing mental health condition who had low levels of support from supervisors (and no control over breaks) were found to have the highest levels of depressive symptoms, while those aged over 45 who rated leadership quality highly were the least depressed group in this study. While older age groups may be suffering less, they appear more willing to seek help and support with serious illness than their younger counterparts [57], which may make identification of arising issues more difficult. These findings further emphasize the importance of factors such as autonomy and relationships associated with the ‘*social’* and ‘*professional’* dimensions of wellbeing [4], and directs organisations to encourage employees to develop regular, meaningful social contact with peers and supervisors; but equally be supported to psychologically detach from work and draw firm boundaries between their work and domestic domains.

### Implications for practice

There is a need to adapt approaches to leadership (and its training) that embrace the differences between home-working and traditional office-based environments and the challenges of ‘virtual’ leadership. It does not seem viable to rely on typical approaches to leadership and management that do not have currency and flexibility in the future work context. Organisations must invest in manager training and adopt a style of virtual leadership that is supportive and empowering (not intrusive or exploitative) alongside clear referral pathways for those needing more professional mental health support. This also raises the opportunity of increasing managers awareness of wellbeing in the workplace, its impact, and strategies for alleviating ill-health and enhancing wellbeing.

### Limitations and future research

Working practices, especially for office-based individuals, are forever-changed. There is a need for research to consider the unique and varied contexts within which employees now work and to apply a range of quantitative and qualitative methods to understand both the ‘what’ and ‘why’ of home-working and its impact on individuals using validated tools [58].

A cross-sectional survey design was chosen for this study due to the ease and speed of implementation in a pandemic context, however the limitations of this design are acknowledged, as is the risk of sampling and survey bias. Though efforts were made to limit this, the analysis is susceptible to random statistical error due to sample size. Equally, the homogenous geographical location of participants must be considered. Nevertheless, this study provides critical insights and direction for future research, which must consider the mediators and moderators of employee wellbeing across larger and geographically diverse groups and provide frameworks for organisations to monitor and evaluate the effect of the workplace, be that office-based, or a blend of both.

## Conclusions

Employee experiences of enforced home-working are influenced by factors such as personality, home environment, access to social support, physical and mental health issues, employment support structures and financial status. Yet, perhaps the most important factor that can be controlled and better managed by organisations is the quality of leadership provided to employees. The Covid-19 pandemic has forced employers to rethink their approach to how, where and when their employees work but the awareness of the need for adapting leadership styles, processes and mechanisms appears to be lagging. There is a need to better understand the factors that positively and negatively influence employee wellbeing and take a more proactive and preventative approach to improving employee outcomes through policy development, manager training and creative health interventions. While the pandemic will pass in time, organisations must consider the impact of future crises on their flexible working practices to build greater resilience in systems and employees. While personal employee factors are not controllable, organisations must develop a greater understanding of the role they play in reducing the likelihood of ill-health and promoting increased wellbeing and subsequently morale and productivity.

## Data Availability

The datasets generated during and/or analysed during the current study are held within Sheffield Hallam University Research Store and are available from the corresponding author on reasonable request.
